# First-principles study on transition metal-doped anatase TiO_2_

**DOI:** 10.1186/1556-276X-9-46

**Published:** 2014-01-28

**Authors:** Yaqin Wang, Ruirui Zhang, Jianbao Li, Liangliang Li, Shiwei Lin

**Affiliations:** 1 Key Laboratory of Ministry of Education for Advanced Materials in Tropical Island Resources, School of Materials and Chemical Engineering, Hainan University, Haikou 570228, People’s Republic of China; 2 Department of Materials Science and Engineering, Key Laboratory of Advanced Materials, Tsinghua University, Beijing 100084, People’s Republic of China

**Keywords:** First principles, Transition metal-doped TiO_2_, Electronic structure, Formation energy, Band edge position

## Abstract

The electronic structures, formation energies, and band edge positions of anatase TiO_2_ doped with transition metals have been analyzed by *ab initio* band calculations based on the density functional theory with the planewave ultrasoft pseudopotential method. The model structures of transition metal-doped TiO_2_ were constructed by using the 24-atom 2 × 1 × 1 supercell of anatase TiO_2_ with one Ti atom replaced by a transition metal atom. The results indicate that most transition metal doping can narrow the band gap of TiO_2_, lead to the improvement in the photoreactivity of TiO_2_, and simultaneously maintain strong redox potential. Under O-rich growth condition, the preparation of Co-, Cr-, and Ni-doped TiO_2_ becomes relatively easy in the experiment due to their negative impurity formation energies, which suggests that these doping systems are easy to obtain and with good stability. The theoretical calculations could provide meaningful guides to develop more active photocatalysts with visible light response.

## Background

The discovery of water photolysis on a TiO_2_ electrode by Fujishima and Honda in 1972 [1] has been recognized as a landmark event. Since then, TiO_2_ has attracted extensive attention as an ideal photocatalytic material because of its excellent properties such as high activity, good stability, nontoxicity and low cost. Thus, it has been widely used in the fields of renewable energy and ecological environmental protection [2-4]. However, as a wide band gap oxide semiconductor (*E*_g_ = 3.23 eV), anatase TiO_2_ can only show photocatalytic activity under UV light irradiation (*λ* < 387.5 nm) that accounts for only a small portion of solar energy (approximately 5%), in contrast to visible light for a major part of solar energy (approximately 45%). Therefore, how to effectively utilize sunlight is the most challenging subject for the extensive application of TiO_2_ as a photocatalyst. In the past decades, many efforts have been devoted to extending the spectral response of TiO_2_ to visible light, including energy band modulation by doping with elements [5-11], the construction of heterojunctions by combining TiO_2_ with metals such as Pt or Pd [12,13] and other semiconductors (such as MnO_2_[14], RuO_2_[15], and WO_3_[16]), and the addition of quantum dots [17] or dyes [18] on the surface of TiO_2_ for better light sensitization.

Because of the unique *d* electronic configuration and spectral characteristics of transition metals, transition metal doping is one of the most effective approaches to extend the absorption edge of TiO_2_ to visible light region, which either inserts a new band into the original band gap or modifies the conduction band (CB) or valence band (VB), improving the photocatalytic activity of TiO_2_ to some degree [19-24]. For example, Umebayashi et al. [5] showed that the localized energy level due to Co doping was sufficiently low to lie at the top of the valence band, while the dopants such as V, Mn, Fe, Cr, and Ni produced the mid-gap states. Yu et al. [21] reported that the density functional theory (DFT) calculation further confirmed the red shift of absorption edges and the narrowing of the band gap of Fe-TiO_2_ nanorods. Hou et al. [22] showed that new occupied bands were found in the band gap of Ag-doped anatase TiO_2_. The formation of these new bands results from the hybridization of Ag 4*d* and Ti 3*d* states, and they were supposed to contribute to visible light absorption. Guo and Du [23] showed that Cu could lead to the enhancement of *d* states near the uppermost part of the valence band of TiO_2_ and the Ag or Au doping caused some new electronic states in the band gap.

Even though the effects of the transition metal-doped TiO_2_ have been investigated frequently, it remains difficult to make direct comparisons and draw conclusions due to the various experimental conditions and different methods for sample preparation and photoreactivity testing. At the same time, because of the lack of the detailed information about the effects of metal doping on crystal structures and electronic structures, there is still much dispute about these issues. In comparison with the experimental investigation, the theoretical analysis by computer simulation can be a proper method to clarify the effects of transition metal doping in detail.

In order to systematically investigate the influence of transition metal doping into anatase TiO_2_, we adopted the planewave ultrasoft pseudopotential method within the framework of density functional theory (DFT) to calculate the electronic structures, formation energies, and band edge positions of supercells, in which a Ti atom was substituted by a transition metal atom. Considering the accessibility of the doping metals, the 3*d* transition metal atoms (*M* = V, Cr, Mn, Fe, Co, Ni, Cu, and Zn) and the 4*d* transition metal atoms (*M* = Y, Zr, Nb, Mo, and Ag) were studied in the present work. Moreover, the present calculation results were compared with the experimental results reported in the literatures. The conclusions are important to understand the reactive mechanism and optimize the performance of TiO_2_ photocatalysts that are active under visible light irradiation.

## Methods

**Figure 1:**
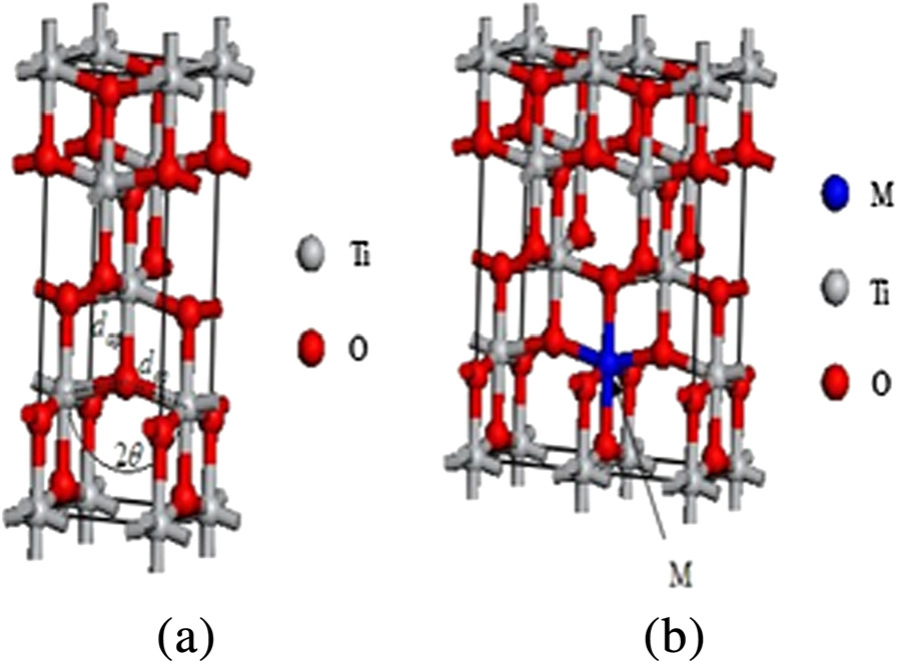
**Models for calculation. (a)** Unit cell of anatase TiO_2_; **(b)** Structure of 2 × 1 × 1 supercell model of transition metal-doped TiO_2_. The gray spheres, the red spheres, and the blue sphere represent Ti atoms, O atoms, and transition metal atom, respectively.

The electronic structures of the transition metal-doped TiO_2_ were studied using first-principles calculation with the supercell approach. The unit cell of TiO_2_ in the anatase structure and the 2 × 1 × 1 supercell model considered in this study are shown in Figure 1a,b. Anatase TiO_2_ has a tetragonal structure (space group, I4_1_/*amd*), which contains four titanium atoms and eight oxygen atoms in a unit cell. Our model consists of two unit cells stacked along the *a*-axes, where one Ti atom was substituted by a 3*d* transition metal atom (*M* = V, Cr, Mn, Fe, Co, Ni, Cu, and Zn) or a 4*d* transition metal atom (*M* = Y, Zr, Nb, Mo, and Ag). The atomic percentage of the impurity was 4.17 at.%.

DFT calculations [25] were carried out using Cambridge Sequential Total Energy Package (CASTEP, Accelrys Company, San Diego, CA, USA) [26,27], with the planewave ultrasoft pseudopotential approach. Our geometry optimizations employed a local density approximation (LDA) exchange-correlation functional, while the Perdew-Burke-Ernzerh (PBE) of the generalized gradient approximation (GGA) was chosen to perform calculations to obtain the electronic structures and accurate formation energies. In these calculations, the cutoff energy of the planewave basis set was 380 eV. The Monkhorst-Pack scheme k-point grid sampling was set as 5 × 5 × 2 for the irreducible Brillouin zone. The Pulay density mixing method was used in the computations of self-consistent field, and the self-consistent accuracy was set to the degree that every atomic energy converges to 2.0 × 10^-6^ eV. The force on every atom was smaller than 0.05 eV/nm. We calculated the total energy and electronic structures in the supercell under these conditions.

## Results and discussion

### Structural optimization

The optimized structures of transition metal-doped anatase TiO_2_ were calculated before the calculations of the electronic structures, which were performed to find the lattice parameters with the lowest energy. As shown in Table 1, the computational results for the structural parameters *a*, *c*, *d*_ep_, *d*_ap_, *c*/*a*, and 2*θ* are summarized together with the reported experimental values [28] and previous theoretical results [29]. The lattice parameters obtained in this work are in good agreement with the experimental data, and the deviation is less than 1.06% along the *a*-axis or 0.5% along the *c*-axis. In comparison with the previous theoretical results reported in [29], our calculation results are more accurate, which verifies that the calculating method and models in this work are reliable and the calculated results are authentic.

**Table 1:** **Optimized structural parameters for anatase TiO**_**2 **_**compared with experimental and previous theoretical results**

	**Experimental**	**This work**		**Literature **[29]	
		**Result**	**Deviation (%)**	**Result**	**Deviation (%)**
*a*/Å	3.785	3.745	-1.06	3.692	-2.46
*c*/Å	9.514	9.466	-0.50	9.471	-0.45
*d*_ep_/Å	1.934	1.914	-1.03	1.893	-2.12
*d*_ap_/Å	1.978	1.969	-0.46	1.948	-1.52
*c*/*a*	2.513	2.528	0.56	2.566	+2.11

### Electronic structure

**Figure 2:**
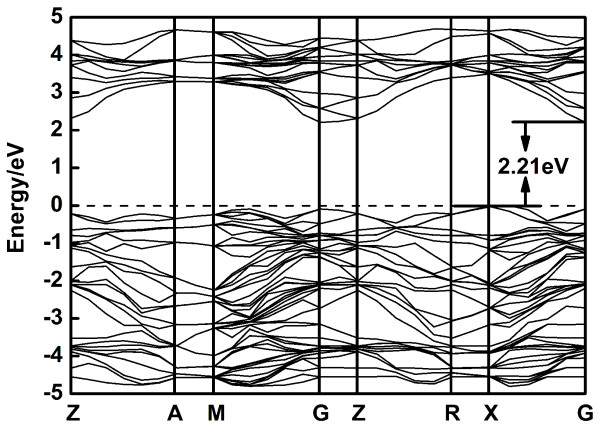
**Calculated band structure of pure TiO**_**2**_**.**

In order to conveniently investigate the electronic structures of transition metal-doped anatase TiO_2_, we set the same k-points mesh to sample the first Brillouin zone for pure and transition metal-doped models. The calculated band gap of pure anatase TiO_2_ is 2.21 eV as shown in Figure 2. The conduction band minimum (CBM) is located at G, while the valence band maximum (VBM) is located near *X*. So, the anatase TiO_2_ can be considered as an indirect band gap semiconductor. The value of band gap is consistent with the reported results [29], but is underestimated compared with the experimental value (*E*_g_ = 3.23 eV), due to the limitation of DFT: the discontinuity in the exchange correlation potential is not taken into account within the framework of DFT. However, our discussions about energy gap will not be affected because only the relative energy changes are of concern.

**Figure 3:**
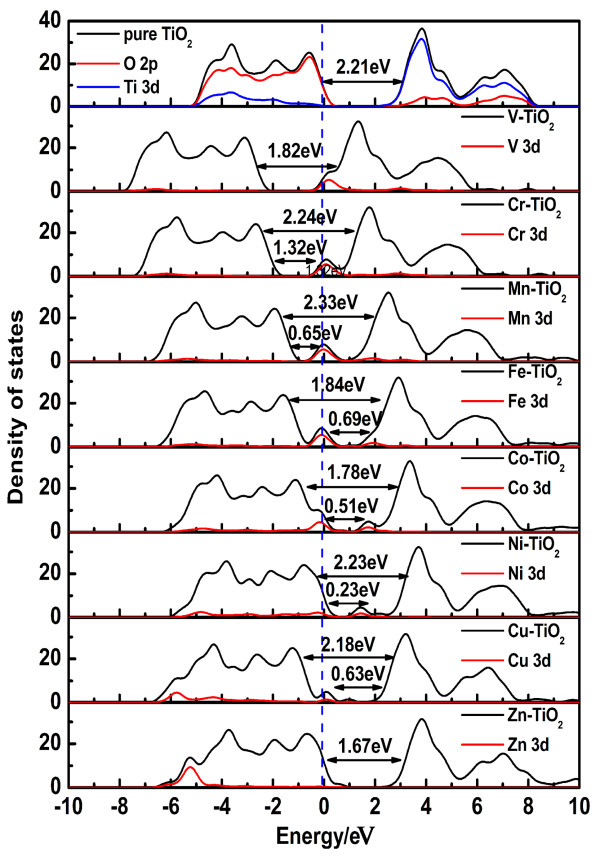
**The TDOS and PDOS of the 3*d *transition metal-doped TiO**_**2 **_**compared with pure TiO**_**2**_**.** Black solid lines: TDOS, and red solid lines: impurity's 3*d* states. The blue dashed line represents the position of the Fermi level.

**Figure 4:**
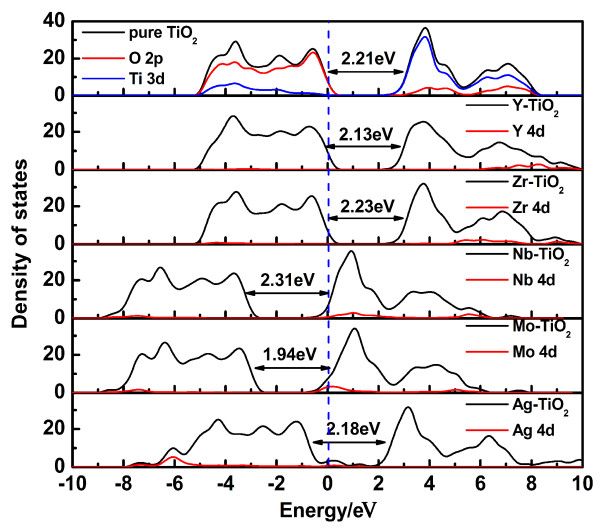
**The TDOS and PDOS of the 4*d *transition metal-doped TiO**_**2 **_**compared with pure TiO**_**2**_**.** Black solid lines: TDOS, and red solid lines: impurity's 4*d* states. The blue dashed line represents the position of the Fermi level.

The total density of states (TDOS) and partial density of states (PDOS) of transition metal-doped anatase TiO_2_ in comparison with those of pure anatase TiO_2_ are shown in Figures 3 and 4, which are treated by Gaussian broadening. The band gap is defined as the separation between the VBM and CBM. The TDOS shape of transition metal-doped TiO_2_ becomes broader than that of pure TiO_2_, which indicates that the electronic nonlocality is more obvious, owing to the reduction of crystal symmetry [19]. The transition metal 3*d* or 4*d* states are somewhat delocalized, which contributes to the formation of impurity energy levels (IELs) by hybridizing with O 2*p* states or Ti 3*d* states. Such hybrid effect may form energy levels in the band gap or hybrid with CBM/VBM, providing trapping potential well for electrons and holes. It gives a contribution to separation of photogenerated electron–hole pairs, as well as in favor of the migration of photoexcited carriers and the process of photocatalysis.

For TiO_2_ doped with V, Cr, Mn, Fe, Co, Ni, Cu, Zn, Y, Zr, Nb, Mo, and Ag, considering the underestimation of the calculations, the band gaps of the transition metal-doped anatase TiO_2_ are corrected by scissors operator. Scissors operator is used for a purpose as correction to the band gap, which has a clear separation between the CB and VB. For these calculations, the scissors operator is set at 1.02 eV, accounting for the difference between the experimental band gap (3.23 eV) and the calculated band gap (2.21 eV) for pure anatase TiO_2_. Then, the band gaps of TiO_2_ doped with V, Cr, Mn, Fe, Co, Ni, Cu, Zn, Y, Zr, Nb, Mo, and Ag, are determined as 2.84, 3.26, 3.35, 2.86, 2.80, 3.25, 3.20, 2.69, 3.15, 3.25, 3.33, 2.96, and 3.20 eV, respectively. It should be noted that the band gap of transition metal-doped TiO_2_ is not related to the band gap between the Ti *t*_2g_ (*d*_*xy*_, *d*_*xz*_, *d*_*yz*_) and *e*_g_ ($d_{z^{2}}$, $d_{x^{2}-y^{2}}$) bands, but to the energy separation between the O 2*p* and the Ti *t*_2g_ bands of TiO_2_ that is modified by doping atoms.

In comparison with pure TiO_2_, the calculation results of the electronic structures of Ti_7_MO_16_ can be classified into six groups according to the position of the IELs in Figures 3 and 4: (1) Ti_7_VO_16_ and Ti_7_MoO_16_; (2) Ti_7_CrO_16_; (3) Ti_7_MnO_16_, Ti_7_FeO_16_, Ti_7_CoO_16_, Ti_7_NiO_16_, and Ti_7_AgO_16_; (4) Ti_7_CuO_16_; (5) Ti_7_ZnO_16_ and Ti_7_YO_16_; and (6) Ti_7_ZrO_16_ and Ti_7_NbO_16_.

**Figure 5:**
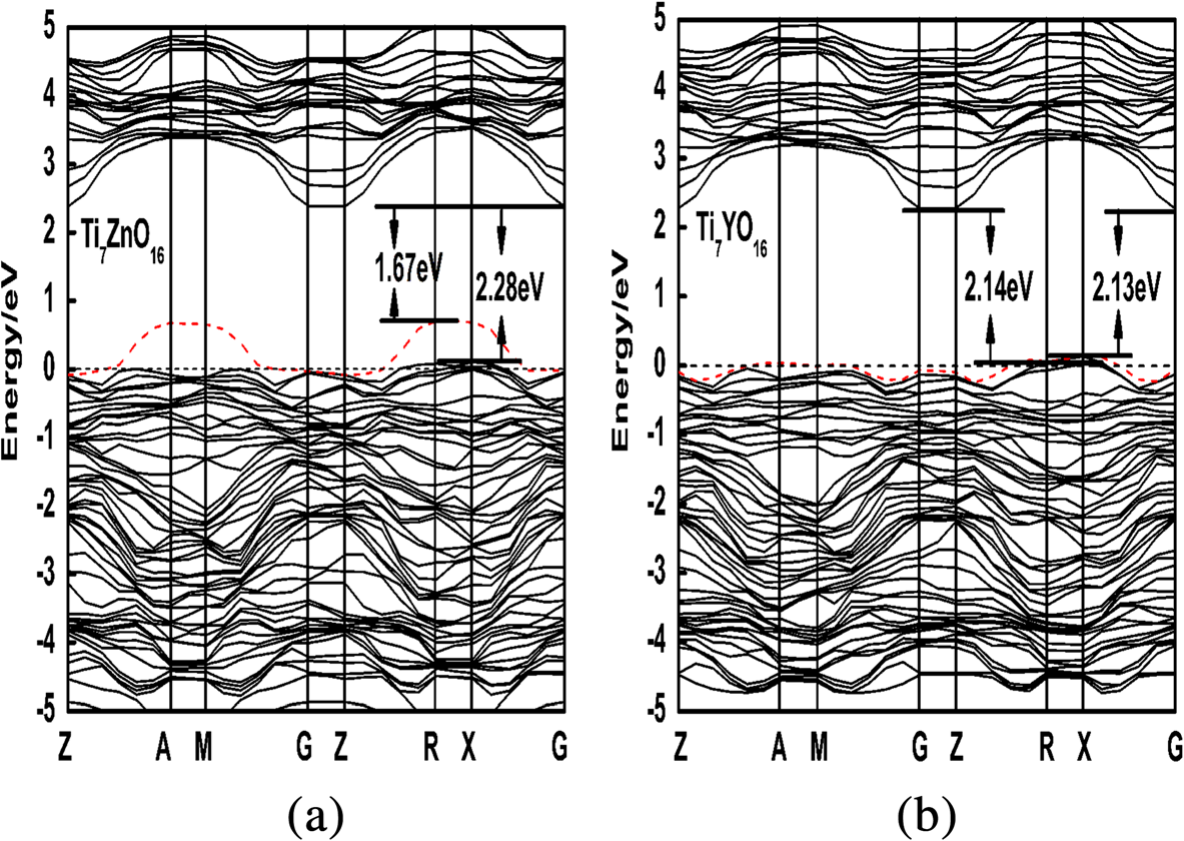
**Calculated band structure. (a)** Zn-doped anatase TiO_2_; **(b)** Y-doped anatase TiO_2_.

• Ti_7_VO_16_ and Ti_7_MoO_16_. The IELs are located at the bottom of the CB and mixed with the Ti 3*d* states to form a new CBM, which leads to an obvious band gap narrowing. The position of the IELs might result in a red shift, which gives an explanation of the experimental optical absorption spectra of V-doped TiO_2_[30]. The positions of the IELs in the Mo-doped system in Figure 4 are similar to those in V-doped TiO_2_, which may also result in red shift of absorption spectra in experiments.

• Ti_7_CrO_16_. The IELs are located below the CBM with a small distance. For Cr-doped TiO_2_, the IELs act as a shallow donor, and their occurrence is mainly due to the Cr 3*d* states that lie at the bottom of CB as shown in Figure 3. As the *E*_F_ crosses it, it is partially filled with electrons at the ground state. In this case, the optical transitions are expected to be two transitions. One is the acceptor transition from the VBM to the IELs. The other is a donor transition from the IELs into the CBM. Meanwhile, VB holes and CB electrons appear. The former contributes to the anodic photocurrent, and the latter contributes to the cathodic photocurrent under visible light. Then, the Cr-doped system can serve as a remarkably better photocatalyst.

• Ti_7_MnO_16_, Ti_7_FeO_16_, Ti_7_CoO_16_, Ti_7_NiO_16_, and Ti_7_AgO_16_. The IELs occur in the middle of the band gap, namely the intermediate level. They may reduce the energy required for electron transition, lower the threshold of photoexcitation, and thus expand the optical absorption spectrum without reducing the energy of electrons or holes. The electrons in the VB can be excited to the IELs and then subsequently excited to the CB by the visible light irradiation. So, IELs are beneficial for extending the sensitive light wavelength. The result gives a good explanation of the red shift [31-34]. However, for these kinds of IELs, high impurity doping concentration might form a recombination center for photoexcited electron–hole pairs and results in a decrease in the quantum yield for the photocatalytic reactions [21]. Therefore, we must control the doping concentration to avoid them to act as the recombination center of photo-generated electrons and holes.

• Ti_7_CuO_16_. The IELs are located above the VB and partially overlap with the VBM. These kinds of IELs could act as trap centers for photoexcited holes, which can also reduce the recombination rate of charge carriers [10]. The holes generated in the VB produce an anodic photocurrent. Because the Cu *t*_2g_ level is close to the VB, the holes easily overlap in highly impure media [5].

• Ti_7_ZnO_16_ and Ti_7_YO_16_. The IELs are located at the top of the VB and completely mixed with the O 2*p* states to form a new VBM (seen in Figures 3, 4, and 5). The band gaps of Zn- and Y-doped anatase TiO_2_ are narrowed to 2.69 and 3.15 eV, respectively, and smaller than that of pure TiO_2_, which is consistent with the experimental data on the red shift of the absorption edge [35,36].

• Ti_7_ZrO_16_, Ti_7_NbO_16_. The IELs are not situated at band gap. The electronic structure of Zr-doped TiO_2_ exhibits similar to that of pure TiO_2_. Therefore, we can infer that the t_2g_ level due to Zr does not contribute to the photo-response. Similarly, the band gap of Nb-doped anatase TiO_2_ is larger than that of undoped TiO_2_ by 0.09 eV, which may result in a blue shift of the absorption edge.

### Formation energy

We analyzed the relative difficulty for different transition metal doping into anatase TiO_2_ using impurity formation energies, which is a widely accepted method. First-principles calculation for the relative stability of metal-doped TiO_2_ can help us understand the formation of the doped structures and provide useful guidance to prepare samples. In this section, to investigate the relative difficulty for single doping atom to incorporate into the lattice, we calculated the impurity formation energy *E*_form_(*M*^q^) as follows [9,37]:

(1) $$\begin{array}{ll} \;E_{\mathrm{form}}\left(M^{q}\right)= & E_{\mathrm{total}}\left(M^{q}\right)-E_{\mathrm{total}}\left(\mathrm{pure}\right)-\sum_{i}n_{i}\mu_{i}\\ & +q\left(E_{F}+E_{v}+\mathrm{\Delta V}\right), \end{array}$$

where *E*_total_(*M*^q^) is the total energy of metal-doped TiO_2_, and *E*_total_(pure) is the total energy of the pure TiO_2_. *n*_i_ is the number of atoms from species *M* (=Ti) being removed from a defect-free cell to its respective reservoir with chemical potential *μ*_i_. The chemical potential reflects the availability or the elemental partial pressure of each element. *E*_F_ is the reference level according to the valence band level (*E*_v_), and *ΔV* is often simplified as zero. In the present work, the transition metal *M* substitutes Ti in the calculated models, and the impurity formation energy *E*_form_(*M*) could thus be defined using the following formula [38,39]:

(2) $$E_{\mathrm{form}}\left(M\right)=E_{\mathrm{total}}\left(M\right)-E_{\mathrm{total}}\left(\mathrm{pure}\right)-\mu_{M}+\mu_{\mathrm{Ti}},$$

where *μ*_M_ is the chemical potential of the doping metal. *μ*_Ti_ is the chemical potential of Ti and depends on the experimental growth condition, which can be Ti-rich or O-rich (or any case in between). Under Ti-rich condition, the Ti chemical potential can be assumed in thermodynamic equilibrium with the energy of bulk Ti, while the O chemical potential can be obtained by the growth condition:

(3) $$E_{{\mathrm{TiO}}_{2}}=\mu_{\mathrm{Ti}}+2\mu_{o}.$$

Under O-rich condition, the chemical potential of O can be calculated from the ground state energy of O_2_ molecule, while the chemical potential of Ti is fixed by Equation (3). The chemical potentials for metals (*μ*_M_) are fixed and calculated from the formula below [40,41]:

(4) $$\mu_{M}=\left({\mu_{M_{m}O}}_{{}_{n}}-n\mu_{O}\right)/m,$$

where ${\mu_{M_{m}O}}_{{}_{n}}$ is the energy of the most stable oxide for doping atoms at room temperature.

The formation energies *E*_form_(*M*) for the 13 different metal-doped models of 24-atom supercell under O-rich condition are calculated and listed in Table 2. In terms of the formation energy, the transition metals that intend to substitute Ti are in the order of Mo < Zn < Ag < V < Y < Cu < Mn < Nb < Fe < Zr < Cr < Ni < Co under O-rich growth condition. It is difficult to find the tendency of *E*_form_(*M*) with the increase in atomic number in each element period. The formation energies of substitutional Co, Ni, and Cr-doped models are negative and less than those of the models substituted by other transition metals under O-rich growth condition. This indicates that under O-rich growth condition, it is energetically more favorable to replace Ti with Co, Ni, and Cr than other metals. The synthesis of the Co-, Ni-, and Cr-doped anatase TiO_2_ system with a higher doping level would be relatively easy in the experiment because a much smaller formation energy is required. This might be because the ionic radii of Cr^3+^, Co^3+^, and Ni^2+^ are close to Ti^4+^. Presumptively, we suggest that the impurity formation energy is sensitive to the ionic radius of impurity. The results can provide some useful guidance to prepare metal-doped TiO_2_ and other oxide semiconductors.

**Table 2:** **Impurity formation energies of 3** ***d *** **and 4** ***d *** **transition metal-doped TiO**_**2 **_**supercells under O-rich condition**

**Metal doping system**	${\mu_{M_{m}O}}_{{}_{n}}/\mathrm{eV}$	***μ***_**M**_**/eV**	***E***_**form**_**(M)/eV**
V/TiO_2_	-6,141.7221	-1,985.7396	1.5761
Cr/TiO_2_	-6,247.8894	-2,472.8718	-0.3744
Mn/TiO_2_	-1,526.5251	-658.4279	1.0589
Fe/TiO_2_	-3,039.9476	-868.9009	0.4044
Co/TiO_2_	-1,478.3064	-1,044.2578	-1.3011
Ni/TiO_2_	-1,789.8414	-1,355.7928	-0.671
Cu/TiO_2_	-1,782.5169	-1,348.4683	1.1586
Zn/TiO_2_	-2,147.2478	-1,713.1992	2.082
Y/TiO_2_	19,299.7106	-3,426.724	1.2848
Zr/TiO_2_	-2,160.6581	-1,292.5609	0.294
Nb/TiO_2_	-19,799.3096	-5,292.2674	0.4089
Mo/TiO_2_	-3,248.3724	-1,946.2266	3.3946
Ag/TiO_2_	-1,462.3681	-1,028.3195	1.77

**Figure 6:**
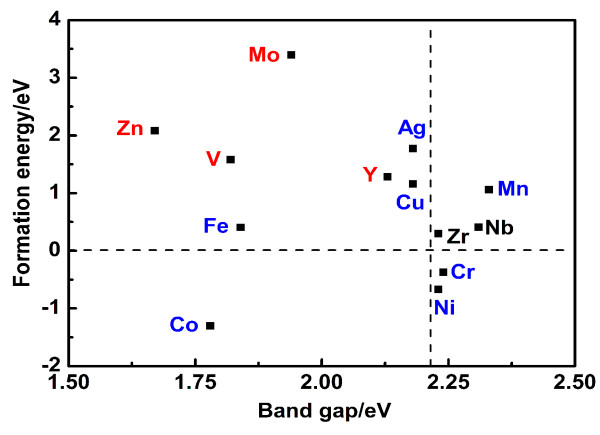
**Relationship between the band gaps and formation energies of 3*d *and 4*d *transition metal-doped TiO**_**2**_**.** The elements colored in black are elements that do not form the impurity levels in the band gap. The elements colored in red are elements that form the impurity levels in the band gap but do not form the middle level. The elements colored in blue are elements that occur in the impurity levels in the band gap and form the middle levels. The horizontal dashed line indicates 0 eV, and the vertical dashed line represents the calculated band gap of pure TiO_2_ (2.21 eV).

To further investigate the influence of transition metal doping, we combine the band gap values and the formation energies of the transition metal-doped TiO_2_ in Figure 6. This can provide important guidance for the experimentalists to prepare thermodynamically stable photocatalysts with visible light response. Under O-rich growth condition, anatase TiO_2_ doped with various transition metals has different formation energies, where the formation energies of Cr-, Co-, and Ni-TiO_2_ are negative. This suggests that such doping is an energetically favorable process. Considering the band gap narrowing effects only, we can find that the band gap is narrowed to 1.78 eV for Co doping, but broadened to 2.24 and 2.23 eV for Cr and Ni doping, respectively. However, TiO_2_ doped with Cr, Co, and Ni, as well as Ag, Fe, Mn, and Cu, which are marked red in Figure 6 and form impurity energy levels in the band gap as shown in Figure 3, might improve the photocatalytic activity with a low doping concentration, but can act as the recombination center for the photo-generated electron–hole pairs with a high doping concentration and result in an unfavorable effect on the photocatalytic activity. In comparison, TiO_2_ doped with V, Zn, Y, and Mo, as shown in Figure 6, possess narrower band gaps than pure TiO_2_ with the IELs mixed with Ti 3*d* states or O 2*p* states. These doping systems result in red shift of absorption edge without forming a recombination center and could improve the photocatalytic activity well. Zr- and Nb-doped anatase TiO_2_ do not form the IELs in the middle of the band gap, and even broaden the band gap, which might result in a blue shift. Furthermore, except for Cr-, Co-, and Ni-doped anatase TiO_2_, the positive formation energies of other transition metal doping systems imply relative difficulty for fabrication in experiments.

### Band edge position

**Figure 7:**
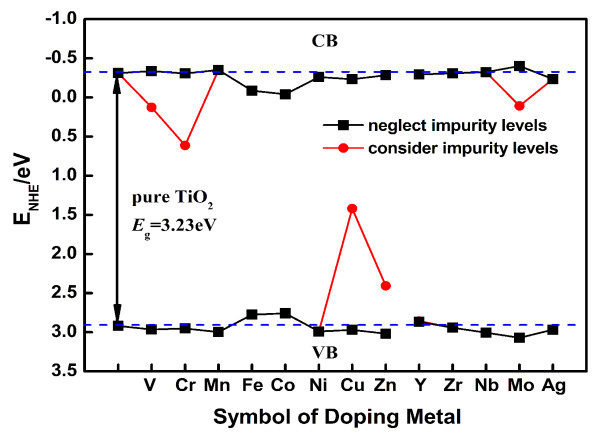
**The calculated band edge positions of 3*d *and 4*d *transition metal-doped TiO**_**2**_**.** The black line is taken as the condition that neglects the impurity levels, and the red line represents the condition that considers the impurity levels. The black line with double arrow is the band gap energy of pure TiO_2_ corrected by scissors operator. The blue dashed lines represent the CB/VB edge potential of pure TiO_2_.

The band edge position of a semiconductor as well as the redox potentials of the adsorbate governs the ability of a semiconductor to undergo photoexcited electron transfer to adsorb substances on its surface [39]. The relevant potential level of the donor thermodynamically needs to be more negative than the VB edge position of the semiconductor in order to donate an electron to the vacant hole. In addition, the potential level of the acceptor is required to be more positive than the CB potential of the semiconductor [42]. So, we calculated the band edge position of the semiconductor photocatalyst to understand the redox reactivity. The CB and VB edge positions of a semiconductor can be expressed empirically by the following formula [43-46]:

(5) ECB=X-Ee-12EgEVB=Eg+ECB,

where *E*_CB_ is the CB edge potential, and *E*_VB_ is the VB edge potential. *X* is the geometric mean of the electronegativity of the constituent atoms [47,48], *E*^e^ is the energy of free electrons on the hydrogen scale (approximately 4.5 eV), and *E*_g_ is the band gap energy of the semiconductor corrected by scissors operator. The CB edge potential of TiO_2_ is -0.31 eV with respect to the normal hydrogen electrode (NHE), while the VB edge potential is determined to be 2.92 eV. This result is consistent with the band edge position of TiO_2_. The band edge positions of TiO_2_ doped with the transition metals relative to that of pure TiO_2_ are summarized in Figure 7, and the data show that most transition metal-doped anatase TiO_2_ can maintain the strong redox potentials. Moreover, in terms of TiO_2_ doped with V, Mn, Nb, and Mo, the CB edges are slightly shifted upward and the VB edges are slightly shifted downward as compared with those of pure TiO_2_. This means that V, Mn, Nb, and Mo doping could even enhance the redox potentials of TiO_2_.

## Conclusions

Transition metal-doped TiO_2_ has been studied using first-principles density functional theory. The calculated results show that owing to the formation of the impurity energy levels, which is mainly hybridized by 3*d* or 4*d* states of impurities with O 2*p* states or Ti 3*d* states, the response region in spectra could be extended to the visible light region. The position of the impurity energy levels in the band gap determines the effects of metal doping on the photocatalytic performance of TiO_2_. Most transition metal doping could narrow the band gap of TiO_2_, lead to the improvement of the photoreactivity of TiO_2_, and simultaneously maintain strong redox potential. Under O-rich growth condition, formation energies of anatase TiO_2_ doped with various metals are different. Particularly, the formation energies of TiO_2_ doped with Cr, Co, and Ni are found to be negative, showing that it is energetically more favorable to substitute Co, Ni, or Cr to a Ti site than other metals. These doping systems can be easily obtained and with good stability.

Theoretical research on transition metal-doped TiO_2_ is of great importance to develop the photocatalytic applications. First-principles calculation of doped TiO_2_ is still an ongoing subject, and a few challenging problems require further investigation in an urgent demand. One is the influence of the transition metal doping on the phase transition of TiO_2_ from anatase to rutile. A theoretical understanding on its mechanism will be useful to optimize the performance of TiO_2_ in photocatalytic and other applications. Another one is the question about using the virtual crystal approximation method to calculate the doping system for very low concentration, which can cut down the calculation time. With the solution of these problems, one could provide more accurate theoretical models to simulate the practical doping approaches which could lead to important implications in the optimization of the performance of transition metal-doped TiO_2_ photocatalysts.

## Competing interests

The authors declare that they have no competing interests.

## Authors’ contributions

SW conceived the idea and designed the calculated model. YQ and RR carried out the calculations and data analysis. JB and LL participated in the design of the study and helped in drafting the manuscript. All authors read and approved the final manuscript.
